# Ischemia/Reperfusion Injury, Its Mechanisms, and Prevention

**DOI:** 10.1155/2012/610370

**Published:** 2012-12-26

**Authors:** Maciej Kosieradzki, Johann Pratschke, Jerzy Kupiec-Węgliński, Wojciech Rowiński

**Affiliations:** ^1^Department of General and Transplantation Surgery, Medical University of Warsaw, 02-006 Warsaw, Poland; ^2^Department of General Transplant and Surgery, Medical University of Innsbruck, 6020 Innsbruck, Austria; ^3^The Dumont-UCLA Transplant Center, David Geffen School of Medicine, Los Angeles, CA 90095, USA; ^4^Department of Surgery, University of Warmia and Mazury, 10-957 Olsztyn, Poland

Since the beginning of transplantation era, ischemia/reperfusion injury (IRI) has remained one of the most serious pitfalls of the procedure. Although thousands of scientific studies and analyses have been published, and mechanisms of the injury recognized and described, the most serious step forward to avoid the IRI was Geoffrey Collins' and Folkert Belzer's invention of two different intracellular-type solutions in 1960s, which allowed for safe renal preservation exceeding 24 hours. Since then, no such important improvement took place and prevention of IRI remains a holy grail of transplantation, especially in thoracic and sub-optimal abdominal organs (fatty livers, older donor kidneys). In this issue we present two studies examining various modalities to reduce both cold and warm ischemia. W. Li et al. showed significant improvement in posttransplantation liver function tests, histology, and reduction of apoptosis with administration of sodium nitrite either to preservation solution or directly to the recipient animal. What is more important is that protection was more evident with more pronounced injury, when liver cold ischemia time was extended to 18–24 hours. Although NO is believed to render protection only in specific concentrations, the authors have shown a dose-dependent effect in a 25–250 uM concentration range. In this edition also, M. G. W. van den Heuvel et al. present use of statins in a human model of approximately one hour warm ischemia and reperfusion during breast reconstruction with free cutaneous flap. Although numerous authors postulate usefulness of statins in attenuation of ischemia/reperfusion injury (IRI) [1, 2], employing dozens of pathways and mechanisms in rendering protection, in this study statins did not show any benefit and rather deteriorated the results and increased risk of complications in skin flap warm ischemia and transposition.

Some agents can be administered in a gaseous form and A. Siriussawakul et al. give a nice overview of potential applications and proposed mechanisms of action of inhaled nitric oxide, carbon monoxide, and hydrogen sulfide in prevention of IRI and inflammation.

Liver is known to tolerate 12 hours of cold ischemia relatively well. However, exceeding preservation beyond this time leads to significant deterioration of both short and long term results of transplantation. This decline is mostly due to biliary pathology, as biliary tree is the most ischemia susceptible tissue of the liver. R. Cursio and J. Gugenheim give a nice overview of biliary complications after liver transplantation. They extensively discuss pathomechanism of ischemic type biliary lesions and the role of cold and warm ischemia as well as intrahepatic cholestasis, bile salts, and immune-dependent events in development of this phenomenon. 

Posttransplant acute renal failure is mainly caused by ischemia and reperfusion. Although primary damage occurs mainly to tubular cells, microvasculature is also affected, especially with prolonged warm ischemia. This becomes clinically more sound with more and more organs retrieved after cardiac death. Patschan et al. discuss features and mechanism of endothelial cells' injury in renal IRI and potential therapeutic application of endothelial progenitor cells. With the focus of contemporary medicine on regenerative technologies this is definitely a “sexy” topic in IRI, regardless of wether peripheral blood-retrieved endothelial outgrowth cells are true progenitor cells or not. What is more important, several authors mention antifibrogenic properties of such therapy, making it even more attractive in the field of renal transplantation. 

A key player involved in inflammation, cellular apoptosis and survival in a transplanted organ is a pathway of mitogen activated protein kinases (MAPKs). Despite its essential role in recovery from ischemia, and some new inhibiting agents with potential therapeutic application in the pipeline, this pathway is rather poorly recognized by transplant physicians. We decided to publish a review by G. Vassalli et al., who give a concise description of MAPK pathway in ischemia/reperfusion injury and ischemic preconditioning of the heart. Bases of this knowledge are critical not only to surgeons involved in heart or other organ transplantation, but equally to any other physician treating patients for coronary disease or myocardial infarction.

*Maciej Kosieradzki* *Maciej Kosieradzki* *Johann Pratschke* *Johann Pratschke* *Jerzy Kupiec-Węgliński* *Jerzy Kupiec-Węgliński* *Wojciech Rowiński* *Wojciech Rowiński*
